# Relations between executive function and emotionality in preschoolers: Exploring a transitive cognition–emotion linkage

**DOI:** 10.3389/fpsyg.2014.00487

**Published:** 2014-05-27

**Authors:** David E. Ferrier, Hideko H. Bassett, Susanne A. Denham

**Affiliations:** Department of Psychology, George Mason UniversityFairfax, VA, USA

**Keywords:** executive function, preschool, emotional expression, emotion regulation, self-regulation

## Abstract

Emotions play a crucial role in appraisal of experiences and environments and in guiding thoughts and actions. Moreover, executive function (EF) and emotion regulation (ER) have received much attention, not only for positive associations with children’s social–emotional functioning, but also for potential central roles in cognitive functioning. In one conceptualization of ER (Campos etal., 2004), processes of ER, and those of emotional expression and experience (hereafter referred to as *emotionality*) are highly related and reciprocal; yet, there has been little research on young children’s EF that focuses on emotionality, although it is easily observed within a classroom. The two goals of the study were to: (1) investigate the relatively unexplored role of emotionality in the development of EF in early childhood and (2) assess the relations between an observational rating of EF obtained after direct assessment with a standardized EF rating scale. We predicted that observed emotionality and EF would both demonstrate stability and predict one another within and across time. 175 children aged 35–60 months were recruited from Head Start and private childcare centers. Using partial least squares modeling, we chose T1 emotionality as the exogenous variable and tested pathways between emotionality and EF across two time points, 6 months apart. Results showed that both T1 observed EF and emotionality predicted their respective T2 counterparts, supporting the idea that both constructs build upon existing systems. Further, T1 emotionality predicted T1 observed EF and the T2 BRIEF-P composite. In turn, T1 observed EF predicted emotionality and the T2 BRIEF-P composite. These findings fit with literature on older populations in which EF and emotionality have been related, yet are the first to report such relations in early childhood. Last, T1 observed EF’s positive prediction of the T2 BRIEF-P composite lends credence to the use of both EF measures in applied and research settings.

## INTRODUCTION

Emotions are thought to play a crucial role in our appraisal of experiences and environments, in guiding our thoughts and actions, as well as regulating our behavior, and in adapting to situations (Cole et al., 2004; Lehtonen et al., 2012). Whereas researchers have started recognizing the interconnections between emotion and cognition, particularly between executive functions (EFs) and emotion regulation (ER; e.g., Blair, 2002; Blair and Diamond, 2008; Blankson et al., 2013), little research with young children has been focused on other aspects of emotion such as emotional expression, even though it is easily observed within a classroom context. In this study, we examine the role of emotional expression and experience (hereafter referred to as *emotionality*) and its interconnection with the development of executive functioning. Before moving to our main questions, however, we should examine the literature already existing on EF and ER.

Executive function and ER abilities have received a large amount of attention for not only their associations with benefits in children’s social–emotional functioning, but also for their suggested critical roles in cognitive functioning (Denham, 2006; Bassett et al., 2012). Moreover, both EF and ER are considered to be aspects of self-regulation (Smith-Donald et al., 2007; Jahromi and Stifter, 2008), which we believe encompasses an individual’s ability to control one’s emotional, behavioral, and cognitive actions and responses (Smith-Donald et al., 2007; Jahromi and Stifter, 2008).

To further define these two aspects of self-regulation, EF is considered a collection of higher-order brain functions, generally viewed as incorporating working memory, attention shifting, and inhibitory control (Miyake et al., 2000; Garon et al., 2008). In terms of its importance, Riggs et al. (2006) wrote of the connections between EF and numerous correlates of social–emotional functioning, such as theory of mind and delay of gratification. Additionally, positive academic achievement outcomes have also been linked to greater EF abilities (e.g., Blair and Razza, 2007; Bierman et al., 2008).

Although different working definitions exist for ER, Campos et al. (2004) chose to define ER as any alteration in the system responsible for the generation and behavioral manifestation of emotions. More specifically, it has been considered “the process of initiating, maintaining, modulating, or changing the occurrence, intensity, or duration of internal feeling states and emotion-related physiological processes, often in the service of accomplishing one’s goals” (Eisenberg et al., 2000, p. 137; see also Thompson et al., 2008). Research has shown that children who have trouble regulating their emotions in the classroom are more prone to exhibit later psychopathology (e.g., Cole et al., 1996), and aggression (e.g., Calkins and Marcovitch, 2010), as well as to suffer from peer rejection, increased anhedonia about school, and poor academic outcomes (Trentacosta and Izard, 2007; Ursache et al., 2012). Further, there is empirical support for the role that ER plays in promoting more positive attributes, such as social competence (Denham et al., 2003) and school adjustment (Herndon et al., 2013).

Clearly both abilities have important sequelae. But how do we view their interrelation? Consistent with the view that ER and cognitive regulation (i.e., EF) are both narrow domains of the broader self-regulation construct (Smith-Donald et al., 2007; Jahromi and Stifter, 2008), Ursache et al. (2012) propose that the connections between the self-regulatory aspects of ER and EF are reciprocal in nature.

Consider the literature on infants which, within the past decade, have both suggested that cognition and emotion are dynamically interwoven (Bell and Wolfe, 2004) and that early indicators of ER positively predicted later EF ability at age four, in children high in emotional reactivity (Ursache et al., 2013). Additional research has provided support for behavioral assessments and parental ratings of inhibitory control in young children concurrently predicting their ER abilities (Carlson and Wang, 2007). Other research investigating parental ratings of ER, suggested that ER supports the later development of EF in preschool-aged children (Blankson et al., 2013). Viewed through a wider lens, findings from studies such as Blankson et al. (2013) and Carlson and Wang (2007) support a transactional model between both EF and ER (Ursache et al., 2012).

These relations are also consonant with developmental neuroscience research, which has also suggested a deeper connection between cognition and emotion centers of the brain (e.g., Cacioppo and Berntson, 1999; Bell and Wolfe, 2004; Carlson and Wang, 2007). Although developmental cognitive neuroscience studies offer suggestions of cognition–emotion linkages, a prevailing notion about the relation between ER and EF suggests that the corresponding areas of the brain connected to these functions are neurologically similar. Calkins and Marcovitch (2010) wrote that empirical connections between EF and ER are, in part, due to areas that are active in the prefrontal cortex (PFC) of the brain. Specifically, two subdivisions within the anterior cingulate cortex (ACC) of the PFC are responsible for cognitive and attentional processes and emotional processes, respectively. In agreement with views from Davidson et al. (2000), Denham et al. (2012a) and Ursache et al. (2012), the model proposed by Calkins and Marcovitch (2010) also suggests that the relations between these two subdivisions of the ACC are reciprocal in nature.

Whatever processes account for this reciprocity, its existence implies that the development, whether typical or atypical, of one aspect of a child’s regulatory capabilities affects the trajectory of other self-regulatory processes. Thus, testing the relations between EF and other aspects of emotion should aid developmental science in understanding equally relevant regulatory processes. In turn, integrating across specific research niches (i.e., EF, ER, and emotionality; Duncan, 2012) can be useful in constructing a more unified knowledge base aimed at preventing specific self-regulatory deficits from cascading across social, emotional, cognitive, and academic domains (see also Blair et al., 2005).

Thus, whereas the interplay between EF and ER is empirically supported within early childhood, the contribution of emotional expression has been overlooked in the self-regulatory literature (Blair, 2002; Riggs et al., 2006; Blair and Razza, 2007; Bierman et al., 2008; Brock et al., 2009). Studies examining cognition–emotion connections have mainly focused on the relation between cognitive and ER (e.g., Calkins and Marcovitch, 2010; Iida et al., 2011); however, a new conceptualization of ER may be what is needed to rectify this limitation of earlier research. In this new formulation, the processes of ER and those of emotionality are highly related, often co-occurring, and reciprocal (Campos et al., 2004). This conceptualization is central to our attempts to address the unanswered relations between EF with emotionality.

More specifically, although the two-factor approach of ER, in which the processes of emotionality and ER are distinguished, has been widely accepted in the past, this model may be an oversimplification (Cole et al., 2004). Instead, uniting emotionality and ER in a one-factor model is a fruitful alternative because it may more faithfully depict the actual process of emotion (Campos et al., 2004). That is, emotions are expressed and experienced almost simultaneously with their regulation; in fact, much of the difficulty in defining and measuring ER lies in its inseparability from emotionality.

Considering the key role that such emotionality plays in ER, then, one would anticipate emotionality, examined uniquely, to also both affect and be affected by the developmental progression of other self-regulatory processes, namely, by an individual’s EF abilities, just as are an individual’s ER abilities. Thus, the overarching goal of the present study is to examine this yet relatively unexplored connection between cognition and emotion: the relation between preschoolers’ EF and emotionality. Finding the relation between EF and emotionality will have a significant benefit not just in research community but also in applied settings. Because, unlike direct assessments of ER that usually involve standard lab procedures eliciting negative emotions from children to observe how they regulate the emotions, emotionality is easily observed in natural settings (e.g., classroom) by preschool teachers.

Based on Campos et al. (2004) unitary process of ER and emotion, we hypothesized that emotionality would be related to the development of EF, and that over time, a reciprocal function between EF and emotionality would be found. Falling in line with the developmental neuroscience literature, we draw additional support for our position from the idea that the more mature portions of the brain responsible for negative emotionality (e.g., amygdala) are capable of inhibiting the deployment, and development, of executive cognitive processes housed in later maturing areas (e.g., PFC; Blair, 2002).

Although research examining the relations between emotionality and EF is scarce with young children, empirical support has been provided for the emotionality-EF link from research with adolescents/young adults. For example, poor EF was found to be related to an increased tendency to express negative affect in college students (Bridgett et al., 2013). In functional neuroimaging research with college students, Luu et al. (2000) also found that affective distress was closely related to frontal lobe EF. If emotionality and higher-order cognitive regulation (i.e., EF) are related in adults, then, examining the relations of these constructs in young children will further aid our understanding of the emotion–cognition interconnectivity from a developmental perspective.

A secondary goal of this paper is to examine the relations between an observational rating of EF obtained after direct assessment with a standardized rating scale. This goal is in order because of difficulties with specificity of EF assessments across age (Best and Miller, 2010). Considerable research has exemplified the range of growth that occurs during the preschool years in young children’s EF (Hughes, 1998; Garon et al., 2008). A common theme amongst prior research was the prediction that measuring EF in preschool-aged children would be difficult due to rapid development, yielding tasks either too easy, resulting in ceiling effects, or tasks too difficult, yielding significantly negatively skewed findings (Hughes, 1998; Isquith et al., 2004; Blair et al., 2005; Carlson, 2005; Garon et al., 2008; Bassett et al., 2012). With the growing notion that inhibitory control and sustained attention not only act as rudimentary forms of EF (Carlson and Wang, 2007; Jahromi and Stifter, 2008; Graziano et al., 2011; Blankson et al., 2013), but also are implicated in the development and utilization of ER, careful measurement and examination of these constructs in a preschool population is of key importance (Riggs et al., 2006).

Two studies have recently contributed to solving this issue of age effects in measuring preschool-aged children’s EF, by using ratings rather than direct assessment. Smith-Donald et al. (2007) developed a two-part assessment of self-regulation, the Preschool Self-Regulation Assessment (PSRA), which is composed of a direct assessment battery and an assessor report (AR) capturing global behavior. The AR consists of several rating items from the Leiter-R social–emotional rating scale (Roid and Miller, 1997) and the Disruptive Behavior-Diagnostic Observation Schedule (Wakschlag et al., 2005). A second study conducted by Isquith et al. (2004) sought to downwardly shift the Behavior Rating Inventory of Executive Function (BRIEF) for a preschool sample (BRIEF-P).

Together, the AR and BRIEF-P have provided measures that do not fluctuate with age as do the more commonly used performance-based tasks, and allow for a more generalizable view of EF. To date, however, there have been no studies looking at relations between the AR and the BRIEF-P. Investigations into their relation could bolster the utilization of rating scales, particularly of scales that are of relative ease of use and do not require a great expense of time.

In sum, research has demonstrated a connection between ER and EF (e.g., Carlson and Wang, 2007). Especially in young children, however, EF’s relation to other aspects of emotion has not been explored. This new unitary perspective on emotionality and ER impels us to consider the heretofore little explored linkage of preschoolers’ emotionality and EF.

In the present study, we collected data using multiple methods and reporters at two time points, to enable us to study relations across short-term longitudinal periods. Specifically, trained research assistants observed children’s emotional expression in naturalistic settings, rated their cognitive regulation (i.e., EF) based on observations of their behaviors during several direct assessments (i.e., social and emotional competence and school readiness), and preschool teachers completed a standardized questionnaire assessing preschoolers’ EF.

**FIGURE 1:**
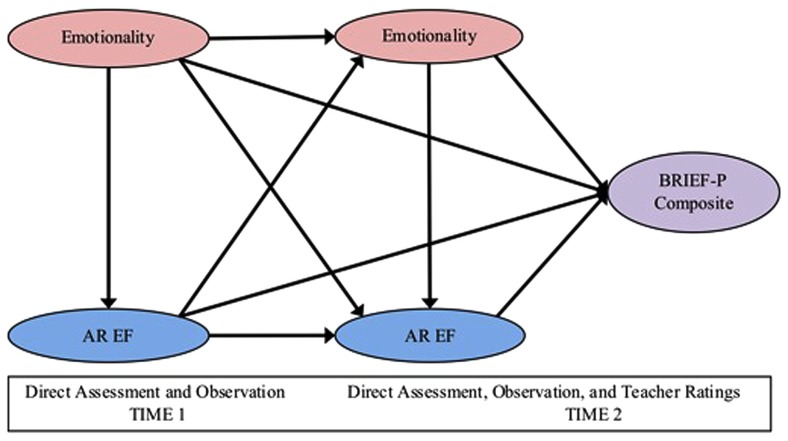
**Partial least-squares outer model**.

Thus, as our first problem question, we examined the relations between emotionality and EF both within and across time in a multi-method approach; we would expect each to show continuity across time, and for emotionality to contribute positively to EF. Although we believe that there is a transactional reciprocity between EF and emotionality, consistent with others (Ursache et al., 2012), with a preschool age sample, we chose emotionality to initially serve as an exogenous variable given that areas of the brain responsible for emotion tend to reach maturation earlier than areas responsible for EF (Martel, 2009; Nigg et al., 2010; Kanske and Kotz, 2012). For this reason, we are testing the directional pathway from emotionality to EF in early childhood within each time period, with cross-lagging pathways between both EF and emotionality between time periods (see **Figure 1**).

Second, given our focus on early childhood development and education, we wished to see how teachers’ views of end-of-year EF were predicted by earlier and concurrent observed emotionality and EF; triangulating across these indices strengthens claims for validity, and thus usefulness, of the teacher ratings of EF in research and applied settings.

## METHOD

### PARTICIPANTS

The current sample is part of a multi-year, multi-site larger project investigating the impact and role that preschool teachers play in facilitating social–emotional competencies. Participants were recruited from ten local Head Start programs and private childcare facilities in the surrounding northern Virginia area, and were culturally, socio-economically, and racially diverse. Children participating were identified via parent contact at recruitment events held at child pick-up, information sessions held at the facilities, and/or through the help of facility social workers and directors.

One hundred seventy-five children aged 35–60 months were recruited for this study and parental consent was attained. Of these, complete data was obtained for 143 (81%) children. Additionally, 36% (*N* = 52) of children were from federally funded Head Start programs. Females comprised slightly more than half of the sample (52.4%). Parents who provided demographic information self-identified as 43.4% Caucasian, 13.9% African–American, 4.9% Asian, 4.2% Multiracial, 3.5% Other; 30.1% of parents did not report their child’s race. Hispanics/Latinos constituted 11.2% of the sample; 28.7% of parents did not report their child’s ethnicity.

### PROCEDURES

Assessments comprised of observation systems and rating scales. Children were assessed in the fall and ~6 months later in the spring. Trained research assistants were either graduate or undergraduate students or volunteers who had extensive training to ensure reliability and appropriate assessment techniques. Because this study is part of a larger grant, additional measures, unrelated to the current study, were administered to participants investigating their social–emotional development. Three direct assessments were administered in a quiet testing environment at the schooling facility at both time points; these measured school readiness, emotion knowledge, and social problem-solving. Following each of these three sessions at both time points, research assistants completed a rating scale about observed EF behavior specific to that session. Additionally, children’s emotionality was observed four times in both the fall and spring data collection periods. Teachers completed a rating scale in the spring session assessing EF in real-world contexts.

To thank the child for participating, a small gift (e.g., small box of crayons or small vial of bubbles) was given to the child at the end of each assessment period. Teachers were compensated $15 for the completion of rating scales for each child.

### MEASURES OF PRESCHOOLERS’ EMOTIONALITY AND EXECUTIVE FUNCTIONING

#### Minnesota Preschool Affect Checklist – Revised/Shortened (MPAC-R/S; Denham et al. 2012b)

The MPAC-R/S is an 18-item observational measure of social–emotional behavior. Previous research has shown that the MPAC-R/S observation system is a valid and reliable tool, with emotionality and regulation related to later preschool classroom adjustment, as well as classroom adjustment and academic success in kindergarten, even age, gender, and prior school success controlled (Denham, 2006; Herndon et al., 2013).

Four 5-min observations were completed by trained observers in both the fall and spring of the academic year and were collected during periods of recess, freeplay, and activity station (“centers”) times. Attempts were made to vary the contexts in which the MPAC-R/S captured data to reduce situation-specific factors from reducing validity. Furthermore, MPAC-R/S sessions were collected on separate days to allow for variability.

In this study, five items were used to specifically focus upon and assess children’s positive and negative emotional expression [e.g., “The child displays positive affect in any manner (i.e., facial vocal, or bodily affect),” and “The child directs negative affect specifically at a particular person when already in contact with them”]; coders take note only of directly observable emotional expressiveness, and, although it is impossible to determine whether any individual child was exerting any internal regulation during any one individual observation period, we feel that by collapsing over several occasions these items are good indicators of emotionality. In analyses to follow, differences in standard scores for positive and negative expression indicated emotionality.

Further, the MPAC-R/S allows for observation of behavioral evidence of ER and dysregulation. Thus, in this study, indices for positive regulation (focusing solely on using language to regulate negative emotion) and dysregulation (focusing on venting outbursts) were also included for subsidiary analyses.

Minnesota Preschool Affect Checklist – Revised/Shortened item content, as well as internal consistency information for the indices of emotionality and regulation/dysregulation, can be seen in **Table 1**. Inter-observer reliability for these data was obtained by calculating averaged measure intraclass correlations (ICCs) for the group of observers, including a master coder. Across two training periods, ICCs were 0.94 and 0.95 for positive emotional expression, 0.97 and 0.98 for negative emotional expression, 0.87 and 0.74 for positive regulation, and 0.98 and 0.99 for dysregulation.

**Table 1:** MPAC-R/S observation items.

Positive emotion (α = 0.77 and 0.67 for T1 and T2, respectively)
1. The child displays positive emotion in any manner (i.e., facial, vocal, or bodily emotion). The child’s behaviors must match the context of a given situation. Examples: smiling, laughing, singing, dancing, etc.
2. The child directs positive emotion specifically at a particular person when already in contact with them. Emotion is directed at a specific person.
3. The child displays positive emotion when in a social situation but does not direct it to anyone in particular.

**Negative emotion (α = 0.92 and 0.93 for T1 and T2, respectively)**
1. The child displays negative emotion in any manner (i.e., facial, vocal, or bodily emotion). The child’s behaviors must match the context of a given situation.
2. The child directs negative emotion specifically at a particular person when already in contact with them. Emotion is directed at a specific person.

**Emotion regulation: positive reactions to emotionally arousing problem situations (α = 0.79 and 0.80 for T1 and T2, respectively)**
1. The child promptly verbally expresses the feelings arising from a problem situation, then moves on to the same or a new activity (versus withdrawing, displacing the emotion onto others or objects, or staying upset).
2. The child shows primarily neutral or positive emotion during this behavior.


**Emotion dysregulation: negative reactions to emotionally arousing problem situations (usually anger-related; α = 0.37 and 0.59 for T1 and T2, respectively)**
1. The child displays context-related interpersonal aggression (verbal or physical). Someone does something emotionally arousing, to which the child responds with aggression (emotionally arousing preceding event must be observed).
2. The child hits, kicks, shoves, knocks over, or throws objects (emotionally arousing preceding event must be observed).

*Average inter-item correlations for all scales were significant (Spiliotopoulou, 2009).*

#### Assessor report

The AR, adapted from a measure originally compiled by Smith-Donald et al. (2007), consists of 12 items asking the researcher to assess the child’s emotional expression, attention, and behavior over the course of an assessment interaction in which data was collected. All items are rated on a 4-point Likert scale ranging from 0 to 3, with five items reverse-coded to reduce acquiescence bias. The AR was administered following direct assessments not in this study at three time points and scores were aggregated to consolidate data into two variables, fall (T1) and spring (T2). Although the AR consists of six scales (Confidence, Affective Balance, Engagement, Attention, Emotion regulation, and Inhibition), only the Attention and Inhibition scales were used in the current study. An example of a prompt assessing Attention was “Distracted by sights and sounds throughout assessment period,” and an Inhibition prompt was “Lets examiner finish before starting task; does not interrupt,” examiners then rate the frequency and intensity from 0 to 3.

In terms of reliability, internal consistency values for the AR factors of Attention (six items) were α = 0.77 at T1 and α = 0.74 at T2, and for Inhibition (three items), were α = 0.54 for T1 and α = 0.61 for T2. Because having a small number of items can negatively impact alpha values, examining the mean inter-item correlations can also provide an accurate representation of internal consistency (Clark and Watson, 1995; Spiliotopoulou, 2009). Mean inter-item correlations for AR Attention were 0.35 at T1 and 0.33 at T2, *p*s < 0.001. For Inhibition, corresponding correlations were 0.29 for T1 and 0.34 for T2, *p*s < 0.001. These values suggest that these items are appropriately related. For inter-observer reliabilities, averaged measure ICC was 0.98 for both Attention and Inhibition.

In terms of validity for the scales utilized here, analyses of the AR by the original authors (Smith-Donald et al., 2007) reported that there were non-significant gender differences, suggesting the presence of construct validity. Furthermore, Smith-Donald et al. (2007) provided concurrent validity for the original AR, showing significant correlations between their Attention/Impulse Control factor and both externalizing and internalizing problems, as well as social competence.

#### Behavior Rating Inventory of Executive Function – Preschool Version (BRIEF-P; Gioia et al. 2003)

Teachers were asked to complete the BRIEF-P at the end of the data collection cycle in the spring of the academic year. The BRIEF-P is a standardized rating scale providing information about the executive functioning of children from ages 2 to 5 years. The measure consists of 63 items providing five distinct scales, one composite scale and three overlapping summary indexes. The BRIEF-P yields five scales assessing Inhibitory Control, Attention Shifting, Emotional Control, Working Memory, and Plan/Organize. These scales reflect all facets of the larger construct of EF and permit comparative benchmarks in EF between subjects. In total, the BRIEF-P takes approximately 10 min to complete.

Excellent internal consistency was found for the five scales (Shift, α = 0.90; Inhibition, α = 0.95; Working Memory, α = 0.95; Emotional Control, α = 0.93; Plan/Organize, α = 0.90). These values were highly similar to the reported values from the test authors (Gioia et al., 2003). Reported validity for the BRIEF-P demonstrated significant correlations across many scales on the Behavior Assessment System for Children – Parent Rating Scales (BASC) with correlations ranging from -0.83 to 0.76 in expected directions.

### DATA ANALYSIS

Partial least squares modeling (PLS: Falk and Miller, 1982; Ringle et al., 2005) was utilized to answer our major problem questions. In common with other modeling techniques, a measurement (outer) model as well as a structural (inner) model is specified. For the outer model, PLS estimates latent variables (LVs) based on the shared variance of the manifest variables, using principal components weights of the manifest variables. As such, each indicator varies in how much it contributes to the LV, resulting in the best possible combination of weights for predicting the LV while accounting for all manifest variables, a distinct advantage of the method (Tsethlikai, 2010).

This method, which is becoming more widely known by developmentalists (e.g., Brody et al., 1994; Cowan et al., 1996; Marjoribanks, 1997; Davies and Cummings, 1998; Isley et al., 1999; Denham et al., 2002, 2003; Bronstein et al., 2005; Tsethlikai, 2010, 2011), also allows exploration of hypothesized relations among constructs without some of the restrictions of LISREL structural modeling techniques. In particular, PLS is appropriate for use with relatively small groups of participants, although it does require a reasonable LV: participant ratio (e.g., 10 times the number of manifest variables for the LV with the largest number of manifest variables, or 10 times the largest number of paths directed at a LV; Henseler et al., 2009). Further advantages include its lack of stringent assumptions such as those regarding observational independence and normality of residuals (Marjoribanks, 1997), as well as error-free measurement (Tsethlikai, 2011).

Outer measurement models provide information on the psychometric reliability of our constructs’ LVs. Inner measurement models do not allow for bidirectional pathways (Barroso et al., 2010), thus, only a unidirectional pathway between LVs was tested within each time point. This estimation assessed predictive validity via the relations among LVs and significant, hypothesized paths. Bootstrapping procedures then allow for significance testing of each path. Further, both inner and outer measurement models provide information on discriminant validity, when LV correlations are compared to the square root of the LV’s average variance extracted (AVE). For this study, LVs are as follows: for both T1 and T2: emotionality and AR EF, and for T2 only: the BRIEF-P Composite. In our model, manifest variables (indicators) were hypothesized to form these LVs, and all hypothesized paths among these LVs were of interest (see **Figure 1**).

## RESULTS

### OUTER MODEL

Using Smart-PLS^TM^ (Ringle et al., 2005), we first examined acceptability of the outer measurement model. Regarding the outer model, three criteria are present: (a) the set of manifest variables represents the same underlying construct (AVE), with a reasonable total explained variance (*R*^2^); (b) the manifest variables also form an internally consistent LV (composite reliability); and (c) each manifest variable loads sufficiently on its LV to support its retention (i.e., each manifest variable contributes to its LV and represents the construct in a similar manner as other manifest variables). According to Henseler et al. (2009), composite reliabilities for all LVs formed by the hypothesized collection of manifest variables should be ≥0.60, and AVE should be ≥0.50. Finally, each manifest variable’s outer model loading should be ≥0.70.

Findings for our model suggested the following (see **Table 2**): (a) all composite reliabilities were >0.60 and (b) all AVEs were >0.50. Further, all manifest loadings were >0.70. Thus, the outer model met all criteria so that inner model analyses could proceed.

**Table 2:** Outer model and final *R*^**2**^s for latent variables.

LV	LV			Manifest loading
Manifest variable	AVE	*R*^2^	Composite reliability	
**Time 1**		–-	–-	
**Emotionality**	1.00		1.00	
MPAC-R/S emotionality				1.00
**Executive function: AR EF**	0.72	0.026	0.84	
AR attention				0.868
AR inhibitory control				0.835
**Time 2**				
**Emotionality**	1.00	0.149	1.00	
MPAC-R/S emotionality				1.00
**Executive function: AR EF**	0.803	0.328	0.89	
AR attention				0.917
AR inhibitory control				0.875
**Executive function: BRIEF-P composite**	0.705	0.150	0.92	
Emotional control				0.819
Inhibitory control				0.876
Planning and organization				0.884
Shifting				0.736
Working memory				0.873

*AVE = Average variance extracted.*

### CONVERGENT AND DISCRIMINANT VALIDITY

**Table 3** shows the square roots of the AVEs and the correlations amongst LVs. This information can yield information on both convergent and discriminant validity. First, for convergent validity, a LV should explain better the variance of its own indicator than that of other LVs. One way to determine this point is to compare the square root of each LV’s AVE with all correlations involving that LV. If the correlation between any two LVs is less than the square root of either of their individual AVE’s, this suggests that each has more internal (extracted) variance than variance shared between the LVs.

**Table 3:** Inner model latent variable correlations.

Scale and time point	1.	2.	3.	4.	5.
1. Emotionality T1	**1.00**				
2. Emotionality T2	0.28*	**1.00**			
3. AR EF T1	0.16^+^	0.31**	**0.85**		
4. AR EF T2	0.05	0.17*	0.57***	**0.89**	
5. BRIEF-P composite T2	0.24*	0.23*	0.32**	0.22*	**0.84**

Square roots of AVEs appear in bold on the diagonal; LV correlations appear below the diagonal. ^+^*p *< 0.10, *p < 0.05, **p < 0.01, ***p < 0.001.

Second, if these criteria are met for a target LV and *all* the other LVs, this suggests the discriminant validity of the target LV (Fornell and Larcker, 1981). Correlations with other LVs of less than |0.7| are also frequently accepted as evidence of discriminant validity. The information in **Table 3** shows that these criteria for both convergent and discriminant validity are met for all LVs in the model. Finally, examination of cross-loadings indicated that each manifest variable’s loading was far higher for its assigned LV than the other LVs; by this criterion as well (not tabled), these LVs showed good discriminant validity.

### INITIAL EVALUATION OF THE INNER MODEL

Given these validity results, we can continue to an examination of the inner model. The first step here is to examine the LVs’ correlations in respect to hypothesized relations among them. As can be seen in **Table 3**, MPAC-R/S Emotionality showed T1 to T2 stability, and both time points’ index of emotionality was related to the BRIEF-P Composite. T2 Emotionality was also related to observed EF at both time points. Finally, AR EF showed T1 to T2 stability, and each time point’s index of observed EF was related to the BRIEF-P Composite.

### OVERVIEW OF STRUCTURAL PATH MODEL

**FIGURE 2:**
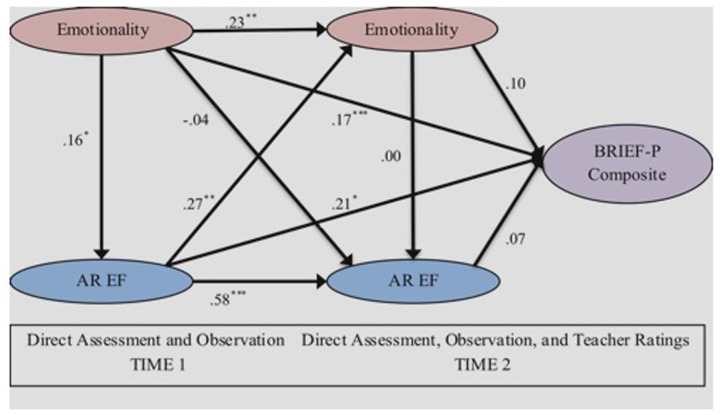
**Partial least-squares inner model.** *Note.* Path coefficients may be interpreted as standardized beta coefficients. Levels of significance determined by *t*-values from bootstrapping procedures and may vary according to the standard error of the path coefficient; *p < 0.05, **p < 0.01, ***p < 0.001.

**Figure 2** depicts the final structural model. Path coefficients in the model can be interpreted as standardized beta weights, each estimated after all other paths’ effects have been controlled. To assess whether the paths were significant, bootstrapping resampling (Efron and Gong, 1983) was performed. In this procedure, the PLS parameters of a series of random subsamples of the total sample are iteratively tested, until significance can be estimated based on their convergent findings.

Our final structural model can be summarized by noting the following significant direct effects of LVs: (1) T1 Emotionality predicted T1 AR EF, T2 Emotionality, and BRIEF-P Composite. T1 AR EF predicted T2 Emotionality, as well as T2 AR EF, and BRIEF-P Composite. T2 AR EF also predicted the BRIEF-P Composite.

### SUBSIDIARY ANALYSES

Two further PLS analyses were undertaken. In the first, several iterations of PLS were attempted. Regulation and dysregulation were included along with emotionality, to show that emotionality was in fact key in the model, rather than merely a marker of regulation. However, outer loadings for regulation and dysregulation in this model did not meet the standard of 0.70 for continued inclusion in the LV. Strict PLS modeling would then require reverting back to the model in **Figure 2**. In these analyses, however, the outer loading for dysregulation, was >0.60 at both T1 and T2, so that a model with emotionality and dysregulation was performed. It was virtually identical for that including only emotionality, suggesting that in fact observed emotionality is key in these analyses. Hence, the primary findings for our research question regarding emotionality and EF, as noted in **Figure 2**, remain.

Second, our research question on how teachers’ views of end-of-year EF are predicted by earlier and concurrent observed emotionality and EF was refined methodologically by deleting the Emotional Control scale from the BRIEF-P LV, to make an even purer EF construct. Again, the PLS model was almost identical to that in **Figure 2**, suggesting that the original BRIEF-P LV, which is based on psychometric standardization of the measure, can be retained for discussion.

## DISCUSSION

### OVERVIEW

This research describes an original endeavor to investigate the relations between emotionality observed in natural settings (i.e., while interacting with peers in preschool classroom) and EF in a preschool population. Conceptualizing ER and emotionality to use the same processes, based on the framework proposed by Campos et al. (2004), we expanded our focus to specifically examine whether relations between EF and emotionality were present as have been found repeatedly between EF and ER. Over time, we believe that emotionality and EF will become reciprocal, a position supported by others (e.g., Blair, 2002). However, given the statistical procedure used and paired with research that has posited that emotion processes develop earlier (Nelson, 1994; Blair, 2002), and in turn influence, more complex cognitive processes, (i.e., EF; Calkins and Marcovitch, 2010; Nigg et al., 2010; Blankson et al., 2013; Ursache et al., 2013), we predicted that measures of emotionality would in turn predict later EF. Using PLS modeling, we were able to test our proposed pathway between emotionality and EF across two time points, approximately 6 months apart; emotionality at T1 predicted observed AR EF at that time, as well as the T2 BRIEF-P Composite. AR EF at T1, in turn, predicted emotionality at T2, as well as the T2 BRIEF-P Composite.

### ANOTHER EMOTION–COGNITION LINKAGE: EF AND EMOTIONALITY

Our primary goal to examine the continuity of EF and emotionality across two time points and to examine the contribution of emotionality to later EF development was supported by our current findings. Subsidiary analyses, (1) including observed dysregulation and (2) excluding the Emotional Control scale from the BRIEF-P LV, did not yield different results from our proposed model. Thus, we are confident to conclude that a significant relation exists between preschoolers’ emotionality and EF. Implications from these findings contribute to the growing literature stressing the importance of emotions in preschoolers’ optimal development (e.g., Denham, 2006; see also Chaplin and Aldao, 2013). Although these findings do not neurologically examine whether portions of the brain dealing with emotion development underlie those areas responsible for EF, the results lend support to previous models detailing their interconnection (Calkins and Marcovitch, 2010; Ursache et al., 2013). Further, this research serves to emphasize that emotionality is implicated in EF abilities, just as ER is often found relating to EF (e.g., Blankson et al., 2013), which suggests that emotionality and ER are part of a larger interconnected self-regulatory network. Finding that T1 scores of EF and emotionality predicted their T2 counterparts also supports the idea that both EF and emotionality are constructs that build upon existing systems (Denham, 2007; Garon et al., 2008). These findings fit with existing literature looking at older populations in which EF and emotionality have also been related (Luu et al., 2000; Bridgett et al., 2013), yet are the first to examine such relations in early childhood.

Thus, the current study contributes empirical support for the promotion of both positive emotionality and EF in preschoolers. In recent times, there have been numerous studies that have separately showcased advantageous outcomes associated with positive emotionality and early precursors of self-regulatory processes, including EF and ER (Denham et al., 2003, 2013; Denham, 2006; Riggs et al., 2006; McClelland et al., 2007; Liew, 2012). Having adequate EF and ER skills and manifesting a more positive emotionality is often considered critical for ensuring numerous positive outcomes, such as school readiness and social–emotional competence (e.g., Denham et al., 2003, 2012a, 2013; Denham, 2006; Trentacosta and Izard, 2007; Brock et al., 2009; Ursache et al., 2012; Herndon et al., 2013). Demonstrating that emotionality contributes to later EF should, we hope, serve to increase the importance of both emotions and EF abilities within the preschool classroom.

Conversely, deficits in EF, ER or more negative emotionality may lead to negative outcomes that could adversely affect numerous facets of optimal development across domains (Denham et al., 2003, 2012a; Denham, 2006; Bassett et al., 2012). This assertion was supported by the current findings, as greater negative emotionality (i.e., indexed by lower or negative emotionality scores) predicted greater EF problems on the BRIEF-P. Through the lens of an educational administrator, these children with greater negative emotionality and/or lower EF would require additional time, effort, and resources from teachers, parents, and supportive staff if problematic behavior were being exhibited.

Developmental researchers are increasingly engaged in addressing and understanding precursors of developmental problems, particularly attention-deficit hyperactivity disorder (ADHD; e.g., Barkley, 1997; Anastopoulos et al., 2011). Children diagnosed with ADHD are marked by lower levels of EF, which have been linked with problems in emotional competence, specifically, ER (e.g., Barkley, 1997; Blair et al., 2005). Understanding early contributing factors to EF will aid preventative literature.

Further, research has shown that exhibiting greater negative emotionality has been strongly linked to numerous poor outcomes, particularly in the preschool and early formal schooling years (Belsky et al., 2001; Denham, 2006; Anastopoulos et al., 2011). Previous research has shown that outcomes such as high ratings of negative behavior by the classroom teacher (Herndon et al., 2013) and lower sociometric likeability and teacher ratings of social competence (Denham et al., 2003) are related to negative emotionality and emotion dysregulation. Recently, a push for preventative practice has underscored the importance of addressing such emotional competence deficits (see also Izard, 2002).

### RELATION BETWEEN THE ASSESSOR REPORT AND THE BRIEF-P

As many teachers are becoming overburdened by high-stakes testing requirements, the utility of easy-to-use assessment measures trumps those that are more laborious and time-consuming. Thus, a second aim of this study was to provide evidence of the BRIEF-P’s usability in research and applied settings. Although rating scales of EF typically manifest low to moderate correlations with direct assessments of the same constructs they are both said to measure, rating scales are less context-specific, averaging the rater’s evaluation of the child over many observations. This property of rating scales has led to the view that they may accurately capture real-world portrayals of EF development (Cairns and Green, 1979; Isquith et al., 2004). Furthermore, the ease of rating scales eliminates the need for extensive training often required by performance-based direct assessments. This study provides support for both the AR and the BRIEF-P, both rating tools assessing EF in preschoolers. Even though the AR requires training, no additional materials are required for its use, unlike direct assessments of EF. Moreover, the AR is an observational measure, not necessitating the direct manipulation of a stimulus set, which translates to a greater flexibility in its applicability. Where there has been limited coverage of the BRIEF-P in settings other than clinical assessment, this study serves to validate its use in more applied settings, such as a preschool classroom or childcare facility. In sum, after demonstrating a significant relation between the AR and BRIEF-P, it is perhaps most useful to choose a specific measure depending on logistical considerations. For instance, the AR can accompany any direct assessor-child interaction, whereas the BRIEF-P offers a less obtrusive approach referencing a broader time frame of behavior.

### IMPLICATIONS FOR POLICY AND PRACTICE

Educators, developmentalists, and policymakers should be informed of the importance of factors such as emotionality and EF for young children, especially those preparing for formal education. Many instances can arise daily, in which children without adequate development in one of these aspects can falter, especially academically and socially (e.g., Carlson and Wang, 2007; Denham et al., 2012c; Herndon et al., 2013). Further, given the plethora of undesirable outcomes associated with low levels of EF and greater negative emotionality in early childhood, it becomes self-evident that the early detection, and addressing, of difficulties in both domains be paramount to promote early social and academic success and school adjustment (Blair, 2002; Denham, 2006; Valiente et al., 2012). Especially because EF are considered to be susceptible to early targeting and interventions (Liew, 2012) and emotional competence can be socialized by preschool classroom teachers (Morris et al., 2013), these results should bolster the ongoing call to arms for curricula and interventions promoting social–emotional learning and EF abilities (Morris et al., 2013; Nix et al., 2013). Further, as this research suggests that both EF and emotionality are related to classroom outcomes, we speculate that the current findings showcase that teachers could find measures potentially useful for predicting positive school outcomes.

### LIMITATIONS AND FUTURE DIRECTIONS

A number of issues exist within the current study, some of which could be addressed in future research. The first limitation to the current findings is that given the structure of data collection, the statistical analyses used required that estimated parameters not be bidirectional. Given prior research (e.g., Carlson and Wang, 2007; Ursache et al., 2012; Blankson et al., 2013), there is reason to believe that during early childhood, a bidirectional effect can be found between EF and emotionality. Thus, despite our belief that a bidirectional relation exists between emotional and cognitive development, we chose emotionality to be our exogenous latent construct. Given a larger sample size, structural equation modeling may be suitable for reevaluating our findings allowing for EF to also predict emotionality at T1. Furthermore, having data from a third time point could also allow for the data to be analyzed for additional bidirectional effects through the use of a cross-lagged autoregressive model, for example. Another limitation is that data was not collected from the parents. Having a third source of data could provide stronger validity to our findings and reduce the possibility that our findings are artifacts of the school environment. Additionally, including parental views on their child’s EF would provide a more representative portrayal of true EF abilities through the inclusion of another context in which young children spend a considerable amount of their time.

Finally, we provide several ideas for future studies. First, collecting neuropsychological data (e.g., fMRI) could provide additional support to corroborate that portion of the brain responsible for emotionality supplement later development of portions in control of EF. Second, although we found support that emotionality positively predicted later EF, it is possible that these effects differ for younger and older preschoolers. We could not begin such investigation because our sample at T2 consisted of more children considered “older” on the BRIEF-P (4:0–5:11) than “younger” children (2:0–3:11). Given the growth that EF undergoes just in early childhood, obtaining a more balanced sample with an equal age distribution could be useful to examine whether the current findings are moderated by age. Although our findings support the idea that EF and emotionality are intricately related, we cannot dismiss the possibility of untested confounding variables. Two variables come to mind: temperament and socio-economic status. The temperament literature highlights a construct, termed “effortful control” that helps in bridging the gap between emotion and cognition (for a brief review, see Liew, 2012; see also Carlson and Wang, 2007). It could be that children high in effortful control are able to display more positive emotionality and greater cognitive control (i.e., EF); it is an avenue that could be investigated in future studies. Family socio-economic disadvantage has also shown to have impact on the self-regulatory abilities of children (e.g., Raver, 2012; Raver et al., 2013) and should also be investigated as another potential confound. Last, in light of the current findings, we implore future research to evaluate the role of emotionality wherever relations are found with ER, adopting the one-factor framework of emotion will allow for a more thorough and comprehensive investigation into the vast domain of self-regulation.

## CONCLUSION

In sum, prior research has evidenced a consistent interrelation between EF and ER. Conceptualizing ER and emotionality as involving unitary processes, this article is one of the first empirical studies to examine whether a similar interrelation exists between emotionality and EF in a preschool population. We hope that our findings, which indicate that emotionality positively predicts later EF, act as a catalyzing agent in understanding the interconnected development of self-regulatory processes. Additionally, we evidenced the use of both observational measures and standardized rating scales as justifiable means of assessing EF skills in early childhood. The acknowledgment of emotionality, which is easily observable within a preschool classroom yet often uninvestigated in the EF and self-regulation literature, warrants future research regarding the implications of early displays of positive and negative affect.

## Conflict of Interest Statement

The authors declare that the research was conducted in the absence of any commercial or financial relationships that could be construed as a potential conflict of interest.
